# Exploring Spanish-speaking patient perspectives on inpatient serious illness communication experiences

**DOI:** 10.1093/geroni/igaf122.4374

**Published:** 2025-12-31

**Authors:** Carine Davila, Heily Chavez Granados, Ana Paola Garza Naveda, Christine Ritchie

**Affiliations:** Massachusetts General Hospital, Boston, Massachusetts, United States; Massachusetts General Hospital, Boston, Massachusetts, United States; Massachusetts General Hospital, Boston, Massachusetts, United States; Massachusetts General Hospital, Boston, Massachusetts, United States

## Abstract

Nearly 3.5 million Americans aged 65 years and older who identify as Hispanic/Latino speak Spanish at home. Compared to non-Latino White older adults, Latino older adults are more likely to have a serious illness, but less likely to engage in advance care planning and serious illness conversations (SIC). It remains unclear what Spanish-speaking older adults view as barriers and factors that impact their SIC experience. To address this, we conducted a qualitative study interviewing 12 Spanish–speaking older adults or their medical decision-makers (mean age 69 years, range 55-95 years, 67% female) who had a documented SIC with a palliative care clinician during a recent hospitalization at an academic medical center. We explored their experiences with SIC with clinicians (not limited to palliative care) and elicited recommendations for improvement then used thematic coding to identify key themes. Participants reported that interpreter interactions with patients and clinicians influence patients’ active engagement in language discordant SIC. Triadic patient-interpreter-clinician interactions can be overwhelming for patients as they simultaneously process emotionally laden medical information and wonder if interpreters are accurately relaying information bidirectionally. In addition, patients and care partners emphasized that faith, spirituality, and cultural background impact their illness experience and care planning (e.g., information-sharing preferences, decision-making preferences, and considerations around potential travel to their home country during illness). These findings indicate that clinicians and interpreters need culturally tailored interventions and personalized approaches to SIC, guided by patient and care partner recommendations, to effectively meet the needs of Latino Spanish-speaking patients.

